# A Study in Nomenclature

**Published:** 1898-12-17

**Authors:** 


### A STUDY IN NOMENCLATURE.

That diseases and the operations devised for their relief should be ticketed with the names of their inventors is perhaps unjustifiable; at any rate, it is a considerable tax upon the memory. Still, the building up of names which shall be anatomically descriptive of the things meant has disadvantages of its own. These are well illustrated by a sentence which we cull from a clinical lecture recently delivered by Dr. Hale White at Guy's Hospital. In describing what is meant by "cholangitis," a word which is coming very much into use, he says it sounds a little uncouth, like most of the terminology connected with the bile passages. Fancy, he adds, how pleased the old woman who liked the word "Mesopotamia" would be if you told her that she had cystic cholelithiasis associated with infective cholangitis, and that if an operation was necessary you could either do cholecystotomy, or cholecystendysis, or cholecystectomy, or cholecystenterostomy, or cholecystolithotripsy, or choledocholithotomy, or choledochostomy, or choledochoenterostomy, or choledocholithotrity, or choledochotomy, or choledochoduodenostomy, or cysticolithotomy. Clearly there is a good deal more in a name than some people imagine.

